# Rethinking parameters of “success” in breaking bad news conversations from patient’s perspective: the successful delivery process model

**DOI:** 10.1007/s00520-024-08354-0

**Published:** 2024-02-22

**Authors:** Martin Koch, Carola Seifart

**Affiliations:** 1 Clinic for Internal Medicine I, University Hospital, Technical University, Dresden, Germany; 2 Research Group Ethics in Medicine, Faculty of Medicine, Philipps-University Marburg, Marburg, Germany

**Keywords:** Breaking bad news, Communication, Cancer, Conversation-model

## Abstract

**Purpose:**

Studies that focus on improving the difficult process of breaking bad news in oncology should include the patient perspective and be guided by appropriate outcome measures. Endpoints such as “patient satisfaction” fall short to capture the complex nature of breaking bad news (BBN) conversations. However, this is true of many studies. The present study attempts to develop a framework model from a new, patient-centered perspective, which can be applied equally in clinical practice and in education.

**Methods:**

Semi-structured in-depth interviews with twelve cancer patients were conducted. Transcripts were analyzed by “qualitative content analysis” following Mayring. Outcomes were further extrapolated in interpretational steps, and a model of “success” from patients view in BBN was developed.

**Results:**

Two central needs of the patients could be identified: *understanding* and *feelings*. Their fulfillment depends on two groups of variables: first, structural characteristics, such as the inevitable *shock*, *individuality,* and *processability*; second, strongly influenceable variables, such as *relationship*, *transfer of knowledge*, and *atmosphere*. From these results, a framework model for successful breaking of bad news from the patient’s perspective was developed: The *successful delivery process* model (SDP model).

**Conclusion:**

As a basic model for the framework for breaking bad news from the patient’s perspective, the SDP model can be applied to many different situations in oncology and help to frame the difficult conversations by tailoring the BBN conversations on determinants that favorably influence the process in a patient centered manner. In this sense, the model can be useful in clinical practice as well as in education.

## Introduction

Breaking bad news is a challenging and complex task for physicians. Receiving a diagnosis of cancer can be psychologically and socially burdening for patients [1–3]; some develop existential fears about their life and future [4, 5]. How a cancer diagnosis is perceived depends on many factors such as patient expectations, prior knowledge [6], and the delivery style of the conveying physician [7, 8]. There is evidence that that the “quality” of communication influences patient-related outcomes such as subjective well-being [9], reduction of anxiety [10], and the decision to continue or stop medical treatment under the guidance of the specialist who had delivered the bad news to them [11]. In addition, bad communication can be medically significant and a source of distress for patients, relatives, and health care professionals (HCPs) [12, 13]. Although communicating bad news is an integral part of an oncologists daily routine, there are only few comprehensive guidelines [14–16], and these have been predominantly developed by “expert opinion” and without patient involvement [17]. Most of the research focuses on patients experience, effects of breaking bad news (BBN), or communication skill trainings [18–20]. A Cochrane analysis about communication skills trainings in cancer found that in only one of 17 analyzed randomized controlled trials (RCT), patients’ perspectives were included when designing the trial’s interventions [21]. It is important to note that scientific evaluation of BBN conversations and communication trainings is mostly conducted through endpoints such as “satisfaction” with the conversation [7, 11, 22–26], reduction of distress and anxiety [27], and participation in decision making [28, 29]. These studies have shown mixed results, with considerable high proportions of dissatisfaction amongst patients [30]. A consensus paper of European experts’ states that cancer patients have to be involved in the definition and further development of reliable and responsive outcome measures [31]. Particular consideration must be given to the endpoints that are chosen, as these are critical starting points for evaluations and outcome measures, both methodologically and in terms of content. The dimension of “satisfaction,” with its generally positive connotation, does not seem to be an appropriate endpoint from patients view, since the delivery of a diagnosis of cancer is by its very nature a deeply negative situation for the persons concerned. Therefore, the present study focuses on the needs of the patients and uses the more patient-centered endpoint of a successful respectively favorable conversation process.

## Methods

### Study design and research question

The aim of the study was to explore cancer patients’ perspectives on “success” in BBN interactions. Since qualitative evaluations are highly dependent on language and etymology of words, our research team decided to use the German term “Gelingen” — best translated with “success,” in the sense of favorable conversation, — as the central expression in our research question and the interview guideline. A qualitative design using semi-structured, individual interviews was chosen to allow patients to reflect their own BBN experience and to state preferences in terms of successful BBN. An interview guideline was designed based on previous research of the authors [32] and a study of existing literature and guidelines.

The study received ethical approval of the institutional review board of the Philipps-University Marburg (ID-No.: 23/14). The study was conducted in accordance with the Declaration of Helsinki, Good Clinical Practice guidelines, and the EU data collection directive. This study is reported following the Consolidated Criteria for Reporting Qualitative Research (COREQ) guidelines [33].

### Setting and patient population

Data collection for this study was undertaken in the outpatient clinic of the department of oncology and hematology of the university hospital of the Philipps-University in Marburg, Germany.

Patients with solid or hematological neoplasms were eligible to take part in the study. There was no limitation regarding stage or therapy of the disease. Patients had to be 18 years or older, had to be fluent in German language. Participants were thoroughly informed about the study, and full written consent was collected before the interviews.

We targeted a purposeful sampling of patients concerning gender, age, formal education, and intention of treatment (curative vs. palliative) to capture a breadth of views. The interviews were continously reviewed throughout the data collection process, in order to monitor data saturation. We reached a consensus in our study group, that data saturation was reached after 12 patient interviews. Patient characteristics are shown in Table 1.

**Table 1:** Characteristics of the patient sample

Characteristic	Number
Sex	
Male Female	7 5
Age	
18–50 50–70 < 70	3 6 3
Relationship status	
Married Widowed/divorced Single	6 3 3
Formal education	
Hauptschulabschluss (basic school qualification) Secondary school High school/university degree	3 3 6
Cancer type	
Hematologic Colon/rectal ENT Lung	5 4 2 1

### Data collection and analysis

All interviews were conducted face to face by MK. The average duration of the interviews was 37 min ranging from 15 to 70 min. The interviews were audio-recorded and transcribed following the rules of the semantic-content transcription [34].

Data analysis was conducted in a three-step procedure, whereas the first two steps followed the paradigm qualitative-content-analysis suggested by Mayring [35], an approach known to be helpful in structuring high volumes of text [36]. In a third step, a model was developed from the data gained from the qualitative-content-analysis.

The analysis was carried out inductively and deductively using the tool MAXQDA 2018. In a first analytical step, a coding framework was developed from three interviews. Important passages in the transcripts were highlighted and reviewed by the research team and clustered into key themes and formed into codes. Some themes and codes emerged deductively from previous studies (e.g., [32]) and known guidelines such as SPIKES, e.g., the fields of information about the disease that are known to be important for cancer patients. The developed code structure was then applied to the remaining interviews and revised in iterative steps using notes and code-memos. Coding was conducted in a collaborative manner, and critical passages were discussed in the research team.

In a second analytical step, the coded passages underwent a cross case analysis in a three-step procedure of (1) reduction — summarizing content of all coded passages, (2) analysis — identifying motifs/phenomena/aspects, and (3) interpretation — comparing the findings of one code between different patients and putting the findings in perspective to the research question.

In a last interpretative step, the model of “successful” BBN was developed from the conclusions of the content analysis.

## Results

Our analysis reflects the complexity of breaking bad news. It is a process that is influenced by a number of factors, and it is of distinct importance for all persons involved. The complex nature has to be considered when trying to determine the meaning of “success” in BBN from a patient perspective. A total of eight main aspects were identified to be important for success in the sense of a favorable communication process. From these factors, we have developed a model for a constitutional framework of successful breaking bad news conversations: the successful delivery process (SDP) model.

### Central needs

The fulfilment of emotional (*feeling*) and informational (*understanding*) needs is central to “success” in BBN: Although patients’ needs are diverse and numerous, emotional support and the need for understanding the given information proved to be essential and central to all patients in the analyzed interviews. However, following the analysis, it is not simply a matter of transporting information and respecting emotional reactions. Therefore, we have conceptually given the needs a broader framework and entitled them “understanding” and “feelings.” Both needs are clearly interdependent and interrelated: the way in which information is passed on and understand, how it can be processed emotionally, or how patients are caught up, depend on each other.

### Understanding

The analysis brings up the meaningful contrast between the deliverer’s viewpoint of “providing information” and the recipient’s perspective of receiving and actually “understanding information.” Not all patients have equal informational needs; these can also change during the course of their disease. Informational needs by patients are highly individual and dependent on the patient’s diagnosis and prognosis, as well as prior knowledge and experience with cancer.

P: (…) I learned in the year of 2012 that there is such thing as a parotid gland. I didn’t know that there was a parotid gland and I didn’t know that it can have cancer. So I really had to get out my biology books. F1, 28

We identified four fields of information needs that could be clustered in (1) diagnosis, (2) prognosis, (3) quality of life, and (4) treatment. Ultimately, figuring out exactly what is important to patients is a challenge in the process. However, in any case, it is not a matter of conveying facts. Rather, it is important to achieve understanding. Understanding refers to the meaning that the bad news and the consequences have for the concrete life situation of the patients. The information must aim to be anchored in the patient’s world.

P: It is really really important to me, what is happening. So first, how is your life going to be. H2, 25

### Feelings

The second central need is the fulfilment of patient’s emotional needs for delivering BBN *successfully*. Being told a diagnosis of cancer often leaves patients with a feeling of great insecurity and uncertainty. Regaining security and overcoming a feeling of isolation, as well as the need for hope were predominant emotional needs that came up in our interviews.

P: The worst is the period of waiting, you are under so much tension, that you don‘t know what to do with your head, your body your feelings, you don’t know what to make of all of it. Your entire life is turned around. (…) F1, 86

Of course, palliative diagnoses in particular are distressing. “Hopelessness” was one of the initial feelings in their experience. We could identify the *feeling of being cared for* and the *building of trustful relationships* to be a substantial emotional need.

P: So, the best possible outcome is, that you trust the doctors that operate, that treat you. Und to know, that they look after you. (…) As a patient it is really important, that you have doctors and (medical) staff around, and that you know that they look after you, that they care for you. F1, 25

Trust can also be reached through *professional expertise* and a high reputation of a hospital or department. Trust can expand from the level of the relationship and help patients to regain lost security in their lives. *Hope* was the other predominant emotional need and a theme that was recurrent in most interviews. Some patients directly acknowledged the balancing act that a hopeful but truthful communication can be for oncologists. Where a curative therapy is not possible, raising hope is especially difficult. In some ways, the various aspects mentioned as necessary for addressing patients’ emotional turbulence and uncertainty are reflected in the expression used by some patients: We need to feel like we are walking the way together.

P: (…) I think it is possible to say – even if someone has a bad prognosis to say: „We can make it!“ Because you are walking this way together. (…) H11, 24

### Influencing factors

Six factors were identified to play a role in achieving the goal of fulfilling patients central needs. Important, these factors differ in the way they can be influenced by persons delivering bad news. Whereas *atmosphere*, *transfer of knowledge*, and *relationship* are largely subject to the influence of the conversation partners*, processuality*, *individuality*, and *shock* are predominantly structural factors that are influenced less directly, but need to be considered and should be reflected in the attitude of physicians delivering bad news.

### Processuality

The “one” breaking bad news conversation was not clearly identifiable in the data, because when talking about BBN, most interviewees referred to a number of consultations. Therefore, *process* is an inherent structural characteristic that has to be considered in breaking bad news. An understanding of BBN as a process is not only an observation but also a request of the interviewed patients. Having more than one BBN conversation was seen as beneficial by most, because it allows to get accustomed with the reality of having cancer. In addition, having an appointment for another consultation felt reassuring and made it possible to understand the large amount of information given to them.

P: It is probably true, that you can’t take it all (the information) in at one push, it is just too much. That means, it is necessary, that the Patient is supported over a period of time and the information is explained to him in bit by bit. H2, 27

### Individuality

Most of the interviewed patients stressed that BBN could only be “successful” if their unique history and *individuality* were taken into account. *Individuality* is a factor that is not influenceable by physicians, and it requires to get to know patients. However, it must be taken into account that the interaction in a conversation is determined not only by the individuality of the patients but also that of the physicians.

P: (…) The problem is that all people are different. And they have different needs. And as I said earlier, it is also important to ask, how the patients want this conversation to be. F1, 15

### Shock

Hearing that they suffer from cancer was a *shock* for most interviewees. The initial feeling after receiving the diagnosis was also characterized as frightening; for some, it was almost physical, as if they “had taken a hit”; one stated that he almost fainted. This deep cut into the reality of life, the shock, seems to be unavoidable and therefore represents a structural element in the delivery of bad news. It has a major (negative) impact on BBN conversations. Patients reported that staying concentrated was almost impossible; some recalled that they were even unable to talk, or to ask questions.

P: When he is saying the word “cancer”, everything that comes after it is gone. (…) Because you are just thinking about the cancer and not the things he says after it. That is the thing. Your head is empty. H7, 31

### Atmosphere

A*tmosphere* was a recurrent theme in the analysis of the interviews. Although a “good” atmosphere is seemingly elusive because it can mean something different to everyone, there are some aspects that can be clearly named: sufficient time, privacy, attention, and appropriate setting.

P: (…) Creating some kind of atmosphere. That would help you to process this shock, that would have been better, than sitting between all that medical equipment with the sliding door opening several times, they had so much to do and other doctors were coming in. H5,5

In all interviews, patients felt that there was “not enough time” for BBN conversations. Some reported that this lack of time caused them not to ask questions because of physician’s busy schedule. This is a dilemma since patients often rely on medicals staff in order to make sense of their disease. Rather than suggesting a minimum amount time, most patients were more focused on the attention received.

P: I think it is more the attention, than the length of the appointment, if somebody is really sincere, a five-minute talk can absolutely be enough. H11, 48

A good *atmosphere* enables the building of relationships and understanding of the disease; it is therefore highly influenceable by medical staff.

### Transfer of knowledge

While understanding is a central need of patients, it naturally emerged that the way in which information is transferred plays an important role. Although it is well known that medical terms inhibit *understanding*, all patients criticized their use.

P: (…) One disadvantage was the attitude of Y, that did not work out well. In principle, he was lecturing in medical terms. (…) That was the deterrent for me, which also moved me to change the hospital. H5, 11

There are three additional recommendations that enable a successful *transfer of knowledge* in BBN conversations from the interviews: provide alternative sources of information, encourage questions by patients and relatives, and ensure understanding by summarizing conversation content.

There is a certain insecurity if you don’t understand everything. This is due to the medical language or to attention. (…) It is really important to understand what is going on. I think it is also the doctor’s job to ensure that the patient understood everything.

I: How can that be done?

P: By asking again…. H11, 80

### Relationship

Qualities like trust, competence, and continuity were of special importance to the participants. Due to the complexity of oncologic diagnoses, many doctors and medical departments were involved in their care. Therefore, most interviewees wanted continuity in the therapeutic relationship over the course of their disease.

P: Naturally (…) the professional competence is very important. (…) but the human aspect is also essential to me, meaning somebody that is caring and communicative and compassionate and empathic, that is of course incredibly important. (…) F1, 38

Most patients stated that they depended on their relatives for understanding complex disease related. Whereby, on the other hand, relatives can also be a burden, especially when patients have to find themselves anew in their role.

## The successful delivery process (SDP)model

The **s**uccessful **d**elivery **p**rocess (SDP)model was created from the factors that were found to be relevant to the successful delivery of bad news. Central are the two needs understanding and feelings. Of course, achieving these goals is a complex process. However, there are factors that are more structural and can be influenced little — individuality, processuality, and shock — and factors that are readily accessible to the influence of the messengers — transfer of knowledge, relationship, and atmosphere. It is precisely the knowledge of these factors, their interaction, and accessibility through the messenger that can contribute to the successful delivery of bad news.

**Fig. 1:**
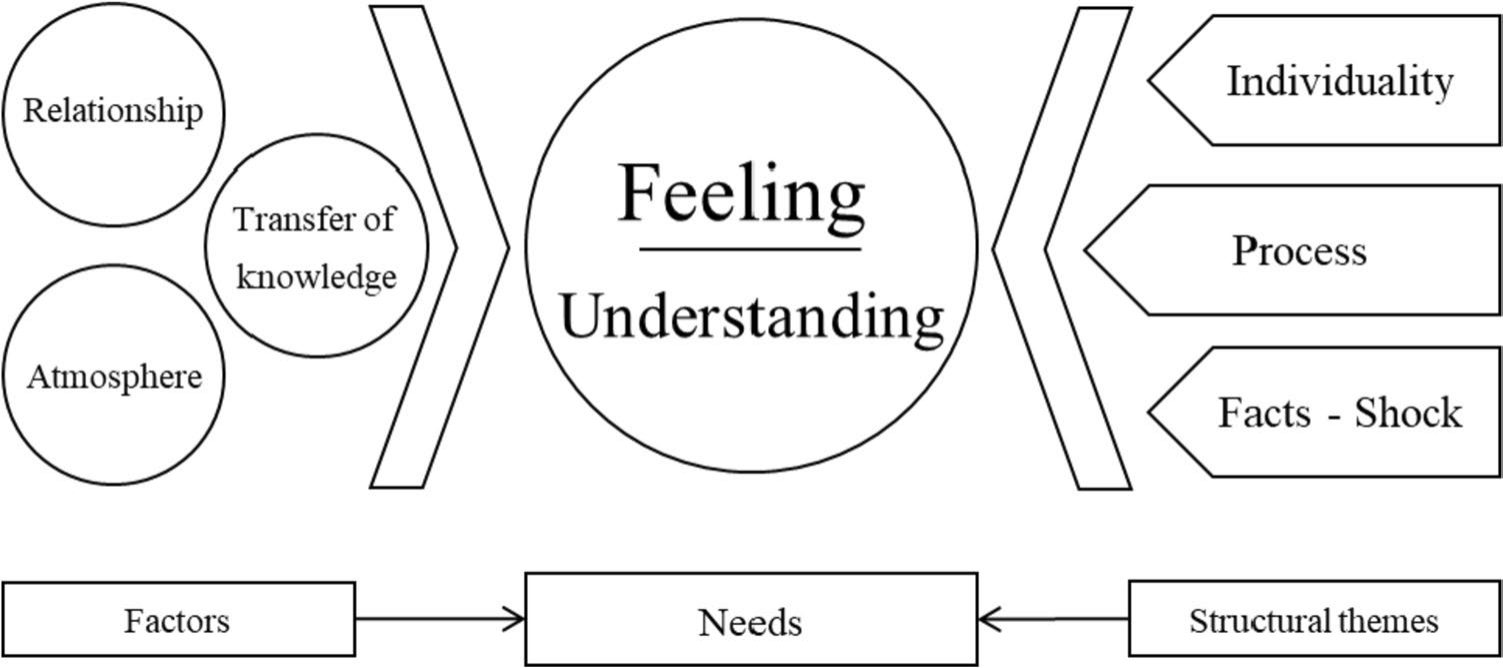
Successful delivery process model (SDP-model) is a framework model designed to facilitate the successful communication of bad news from the patient’s perspective: In order to support the patient’s central needs (feelings and understanding), there are structural factors (left side: individuality, facts/shock, process) that can be influenced only minimally by the messenger, but nevertheless play a crucial role for the course of the conversation. On the other hand, there are factors that are strongly influenced by the messenger (right side: relationship, atmosphere, transfer of knowledge)

The model is illustrated in Fig. 1.

## Discussion

Delivering bad news is a challenging task for physicians, which by its very nature has a “bad” and burdening meaning for patients. Consequently, the conversation process is also difficult to evaluate in positive connotated parameters such as satisfaction. This may also be a reason why a number of studies measured a low satisfaction of patients [11, 32]. However, it is also impossible to avoid having breaking bad news conversations and the purpose associated with it. From the patient’s point of view, therefore, a favorable course of conversation is a more appropriate endpoint. In this study, therefore, with this endpoint in mind, patients’ preferences for breaking bad news were identified.

Clearly, breaking bad news is an extremely complex process that depends on many different factors (communication skills, attitudes, situation, context, circumstances etc.). The present framework model is designed to go beyond individual sub-aspects to achieve a more global, processual understanding from a patient-centered perspective. Therefore, the model has several advantages. It provides an overview of the factors that influence a favorable conversation process without specifying individual procedures. The model can therefore be applied to a wide range of clinical situations. Unlike other guidelines or models, this model assumes that there are certain conditions and/or effects that can be influenced only slightly, while others are more under the control of physicians.

Following the SDP-model a favorable conversation process depends on reaching patients’ emotional and informal needs. Satisfying an existing need for information is a well-known and necessary goal when delivering bad news [17, 37]. In most cases, similar expressions such as “informational needs adequately addressed” are used [38]. However, what this means exactly is not always clear, especially when considering that different patients need different information. From the perspective of “successful” BBN, the information should be passed on in a way, that patients gain an understanding of their (new) situation in the context of their life. Whereby “understanding” is meant in a fundamental and not in a pure transport-of-information sense. The information must be anchored in the patient’s lifeworld. This is achieved, on the one hand, through linguistic appropriateness, which is known to be an important prerequisite for delivering bad news [11, 39, 40], but also through concrete orientation to the reality of the patients’ lives themselves and values [13]. Informational and emotional needs are closely linked and influence each other. It is well known that emotional, psychological, or holistic support is required when delivering bad news [20]. The bad news shakes the patient’s world and puts them in a state of great uncertainty. Therefore, regaining security, emotionally and cognitively, is crucial for a successful BBN process. Previous qualitative studies have identified the phenomenon of “existential uncertainty” in the context of cancer. There are indications that patients receiving palliative care suffer from “the struggle of living with an uncertain future” [41]. Others have described the *shock* of the diagnosis as a biographical disruption [42]. This biographical disruption and a certain degree of uncertainty seem to be independent of the way bad news are communicated.

Some of these factors are known from other guidelines or communication models [21, 24]. However, the SDP-model makes the connections from a patient-centered perspective for the needs of patients and identifies the possible adjusting screws for practice.

### Practical implications

Breaking bad news is a balancing act that requires oncologists to constantly adapt to various factors and circumstances. Precisely, because the SDP-model outlines the framework for a favorable conversation process when delivering bad news. It enables a fundamental understanding and is applicable to a wide range of diverse clinical situations. Understanding what factors can contribute to a need-based communication process allows this difficult challenge to become more patient-centered. The SDP model is therefore also ideally suited for teaching and training of future pysicians and health care professionals. The model is versatile and adaptable to other cultural contexts and other medical fields other than of oncology.

### Limitations

Owing to its qualitative nature, this study does not provide representative data how cancer patients think about “success” in BBN conversations. Because of the methodological approach, the analyses depends on preliminary (linguistic) considerations. Furthermore, the interviews were conducted with white Caucasians only. The authors are aware that German society is more diverse and that the studied sample is not an accurate representation of it. In the complexity of breaking bad news, sociocultural background plays an important role, although the present study considers only a small sample of it. Further studies should take these aspects into account and further evaluate the model in an application-related design.
